# Building Capacity for Community-Engaged Research: Frameworks and Methods for Successful Aging Science

**DOI:** 10.1093/geroni/igaf122.711

**Published:** 2025-12-31

**Authors:** Liza Behrens, Ally Brothers, Cal Halvorsen

## Abstract

Capacity building is integral to the success of community engaged research (CEnR). This is especially important for collaborating with new community and research partners. With the rapidly changing healthcare environment, researchers are often challenged to build capacity for successful research with older adults and their communities. This symposium will explore innovative frameworks and methodologies for capacity building in CEnR, focused on dementia care and healthy aging outcomes. The session will feature four presentations that highlight different approaches to fostering capacity development and community engagement in diverse settings. The first paper will present a framework for capacity development in Villages, a community-centered model for healthy aging. This research identifies key thematic categories for capacity growth, including understanding and motivation for research, cultivating partnerships, theorizing interventions, and procuring data systems. The second paper integrates CEnR and Two-Eyed Seeing, combining biomedical and indigenous knowledge frameworks to create a comprehensive dementia evaluation toolkit for Indigenous populations. The third paper shares insights on how capacity building using CEnR frameworks can drive sustainable changes in a significant public health challenge. The fourth paper highlights the barriers and challenges encountered while implementing CEnR frameworks and offers valuable lessons on what not to do in similar initiatives. The session will conclude with a moderated discussion that will engage presenters and participants in exploring strategies to advocate for community engagement in aging science, reports to funders, and plan for future capacity building work with community partners.

